# Very limited usefulness of pulse pressure variation as a predictor of volume responsiveness in critically ill septic patients

**DOI:** 10.1186/cc13336

**Published:** 2014-03-17

**Authors:** GG Gavriilidis, IK Kostakou, VT Tsagari, AK Kyriakoudi, NK Koulouris, AK Koutsoukou

**Affiliations:** 1 University of Athens, Sotiria Chest Hospital, Athens, Greece

## Introduction

The aims of the study are to assess the usefulness of pulse pressure variation (PPV) as a predictive marker of fluid responsiveness and to estimate the value of central venous-arterial difference of carbon dioxide (PCO_2_cv-a) to predict the outcome of critically ill septic patients. The question of whether a septic patient needs fluids or not is crucial. Although PPV is a very reliable predictor of volume responsiveness, there are many limitations for its application. Cardiac arrhythmia, spontaneous breathing and low tidal volume ventilation prevent the extended use of this index.

## Methods

This is a *post-hoc *analysis of data from a prospective observational study [1], which included a population of patients with severe sepsis or septic shock as the main reason for ICU admittance. After an echo for the assessment of the cardiac systolic performance, we measured PPV, central venous oxygen saturation, blood gases from arterial and central venous lines, central venous pressure, systolic, diastolic, mean and pulse pressures, before and after volume challenge. We calculated changes in pulse pressure before and after volume challenge, and an increase of 9% was used as criterion to define volume responsiveness [2].

## Results

Among 72 patients (71% men, APACHE II score 23.2) included in this study, 41 (57%) were responders. Due to spontaneous breathing and cardiac arrhythmia we were able to calculate PPV only in 18 patients (25%). Moreover, only eight (11%) calculations of PPV proved useful because of the application of low tidal ventilation (4 to 6 ml/kg ideal body weight) in the remaining 10 patients. We did not find any value of the PCO_2_cv-a to predict the outcome of the patients (mortality 26.3%), since the difference between survivors and nonsurvivors was not statistically significant (7.7 vs. 7.9, *P *>0.05).

## Conclusion

The usefulness of PPV, the marker with the best performance in the prediction of volume responsiveness, due to its limitations, is very limited.
